# Minoxidil induced hypertrichosis in a 2 year-old child

**DOI:** 10.12688/f1000research.2-226.v1

**Published:** 2013-10-28

**Authors:** Ingrid Herskovitz, Joshua Freedman, Antonella Tosti

**Affiliations:** 1 University of Miami Hospital South Building, Miami, FL 33136, USA

## Abstract

We report a case of a 2 year-old male patient who developed generalized hypertrichosis after 2 months of treatment with 5% minoxidil foam for alopecia areata. This report highlights the danger of prescribing  topical minoxidil to young children and the need to correctly instruct caretakers about its administration.

## Case

Topical minoxidil is widely utilized as an off-label therapy for alopecia areata in adults and children. We report here a case of generalized hypertrichosis in a 2 year old child.

The patient was a 2-year old hispanic boy with no other significant medical history, who was affected by patchy alopecia areata involving 40% of the scalp since the age of 1 year. Three months before the patient came to our clinic he was prescribed 5% minoxidil foam to be applied to affected areas of the scalp twice a day. After two months the parents noticed hair regrowth but also growth of long pigmented hairs on his face, trunk and limbs. The patient’s mother admitted that she had possibly been applying more product than originally instructed.

**Figure 1.**
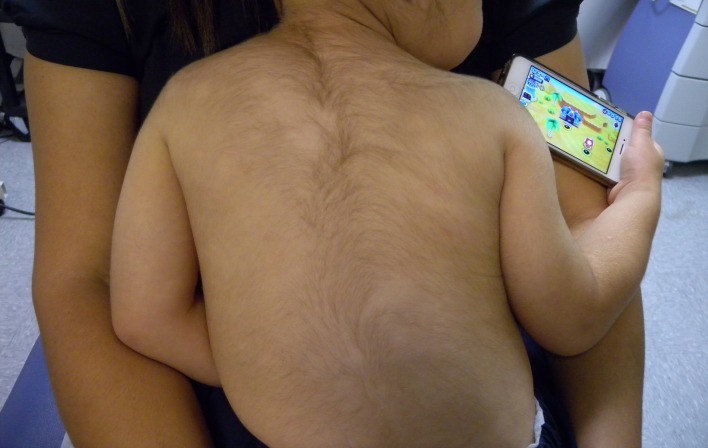
Note the augmented number and thickness of hairs in the back and face of this young child caracterizing hypertrichosis.

Clinical examination showed patchy alopecia areata involving 10% of the scalp and generalized hypertrichosis ( Figure 1). No other side effects were observed. The patient was referred to a pediatric endocrinologist who excluded underlying endocrinological abnormalities. Minoxidil was discontinued and considerable clinical improvement of the hypertrichosis and the scalp alopecia areata was observed at two month follow up.

## Discussion

Minoxidil affects hair growth through incompletely understood mechanisms; known effects include increased duration of the anagen growth phase, agonistic affects on adenosine-triphosphate (ATP)-sensitive potassium channels, and prostaglandin stimulation in the dermal papillae. It is clinically indicated as a therapy for androgenetic alopecia, however off-label uses include topical application in alopecia areata in both adults and children.

Systemic administration of minoxidil either by oral administration to the mother during pregnancy or by oral ingestion by the child, has led to rarely reported instances of diffuse hypertrichosis in children ^1, 2^ and newborns via maternal–fetal transmission ^3, 4^.

Hypertrichosis is a common side effect of topical minoxidil treatment in women. Although usually localized to the face, it may occasionally involve limbs and other body areas ^5, 6^. To our knowledge there are no reports of generalized hypertrichosis in a pediatric population.

Systemic absorption of the drug is typically minimal with topical therapy, with 1.4% of the applied dose being absorbed ^7^. However, hypotheses on the pathogenesis of the diffuse hypertrichosis reaction routinely include systemic absorption, as well as high sensitivity of the follicular apparatus to minoxidil ^5^. In our patient, the excessive dose (both in terms of concentration and daily quantity) in combination with the patient’s low body weight favoured systemic adsorption. Further support for systemic effects are noted in the reported cardiovascular side affects in three patients from 10 to 14 years of age treated for alopecia areata with minoxidil 2% topically twice a day ^8^. These effects included sinus tachycardia, sensation of palpitation and dizziness.

## Conclusion

The efficacy of topical minoxidil in alopecia areata has never been definitively proven ^9^. The possibility of systemic absorption contraindicates, in our opinion, this treatment in young children, who can develop serious cutaneous or systemic side effects. Furthermore, there are some alternative treatments of alopecia areata in children that are considered safer, for example topical immunotherapy and topical anthraline application.
