# Supplementary material for: Minoxidil induced hypertrichosis in a 2 year-old child
Source: F1000Res. 2013 Oct 28;2:226. [Version 1] doi: 10.12688/f1000research.2-226.v1 (PMC3886784; doi:10.12688/f1000research.2-226.v1)
Supplement: Peer review history

# Peer review: Referee response for version 1

**Published:** 2013-11-11

**Authors:** Johannes Kern

**Affiliations:** 1 Department of Dermatology and Venereology, University Medical Center Freiburg, University of Freiburg, Freiburg, Germany

This is a well-written and instructive case report. It emphasizes that minoxidil, which is not a standard first line therapy in alopecia areata, should probably not be used in young children because of possible side effects.

Some comments:

1. Detailed information about the endocrinological work-up performed would be of interest.
2. Were minoxidil levels in the blood of the child assessed?
3. Was there a family history of hypertrichosis?
4. Is there a follow up picture that shows the improvement of the hypertrichosis after discontinuation of minoxidil?

I have read this submission. I believe that I have an appropriate level of expertise to confirm that it is of an acceptable scientific standard.

# Peer review: Referee response for version 1

**Published:** 2013-11-05

**Authors:** Mohamed Badawy Abdel-Naser

**Affiliations:** 1 Faculty of Medicine, Ain Shams University, Cairo, Egypt

This case report is well written and informative. Here are a few comments:

1. " *The patient was referred to a pediatric endocrinologist who excluded underlying endocrinological abnormalities*."

- It would be very helpful if a short paragraph about the possible endocrinologic causes and the lab work done by the endocrinologist to exclude them could be added.

2. " *Minoxidil was discontinued and considerable clinical improvement of the hypertrichosis and the scalp alopecia areata  was observed at two month follow up*"

- Are there any photographs to demonstrate the improvement of hypertrichosis?

I have read this submission. I believe that I have an appropriate level of expertise to confirm that it is of an acceptable scientific standard.
